# Supplementary material for: UniScore, a Unified and Universal Measure for Peptide Identification by Multiple Search Engines
Source: Mol Cell Proteomics. 2025 Jun 2;24(7):101010. doi: 10.1016/j.mcpro.2025.101010 (PMC12272588; doi:10.1016/j.mcpro.2025.101010)
Supplement: Supplementary Material [file mmc1.pdf]

Supplemental Data

UniScore, a unified and universal measure for peptide identification by multiple search engines

Tsuyoshi Tabata[1], Akiyasu C. Yoshizawa[1], Kosuke Ogata[1], Chih-Hsiang Chang[2], Norie Araki[2], Naoyuki Sugiyama[1,3], Yasushi Ishihama[1,4]*

1) Graduate School of Pharmaceutical Sciences, Kyoto University, Kyoto 606–8501, Japan
2) Graduate School of Medical Sciences, Kumamoto University, Kumamoto, 860-8556, Japan
3) Omics Research Center, National Cerebral and Cardiovascular Center, Suita, Osaka 564-8565, Japan
4) Laboratory of Proteomics for Drug Discovery, National Institute of Biomedical Innovation, Health and Nutrition, Ibaraki, Osaka 567-0085, Japan

*Corresponding author: Yasushi Ishihama, yishiham@pharm.kyoto-u.ac.jp

---

Table of contents

Figure S1. UniScore calculation

Figure S2. Base-peak chromatogram of a HeLa global proteome sample.

Figure S3. Correlation between UniScore and Mascot ion score for peptides with different physical properties in Files 1-5.

Figure S4. Effect of charge conversion on UniScore of peptides with different precursor charges. The red dotted line shows y = x.

Figure S5. Decoy-based FDR using linear discriminant analysis is well-calibrated against entrapment-based FDP.

Table S1. Datasets and data analysis parameters used in this study.

Table S2. Effect of product ion abundance filtering on UniScore-PSM at FDR 1%.

Table S3. Effect of matched b-, y-ions on UniScore-PSM at FDR 1%.

Table S4. UniScore-based PSMs at FDR 1% (target-decoy) and FDP 1% (entrapment).

Table S5. Various Search Engines, UniScore and Quantms for PSM at FDR 1%.

Table S6. Estimation of UniScore Calculation

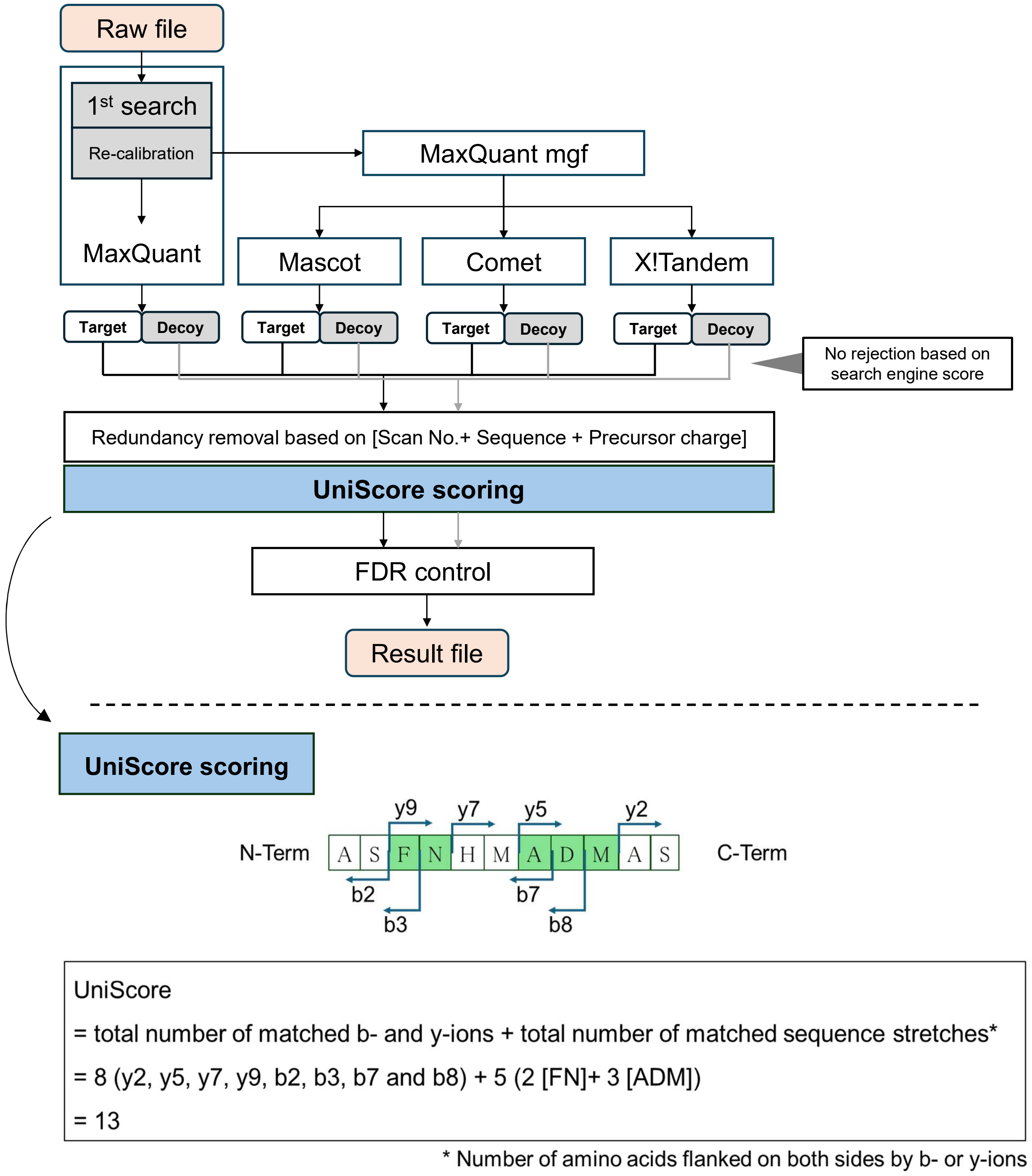

Supplementary Figure S1: UniScore calculation

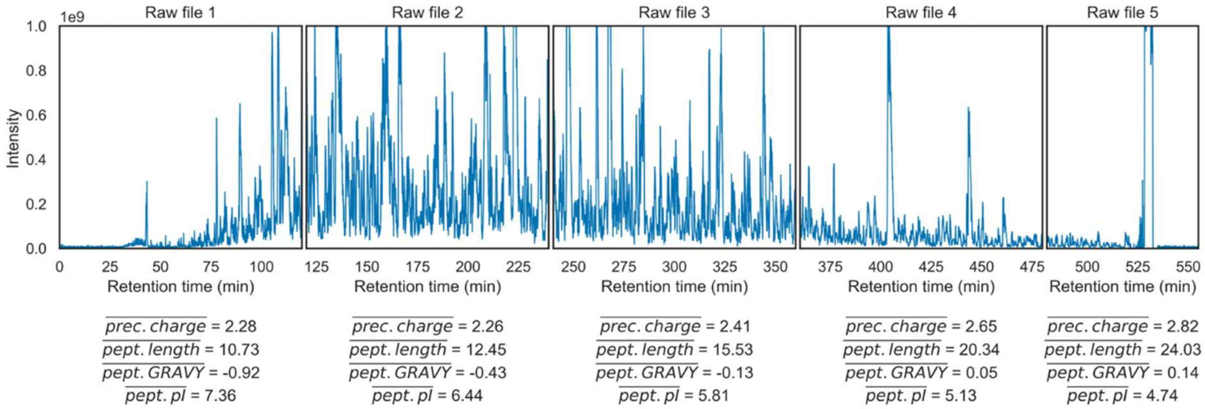

Supplementary Figure S2: Base-peak chromatogram of a HeLa global proteome sample. Data was obtained from PXD005159/JPST000200.

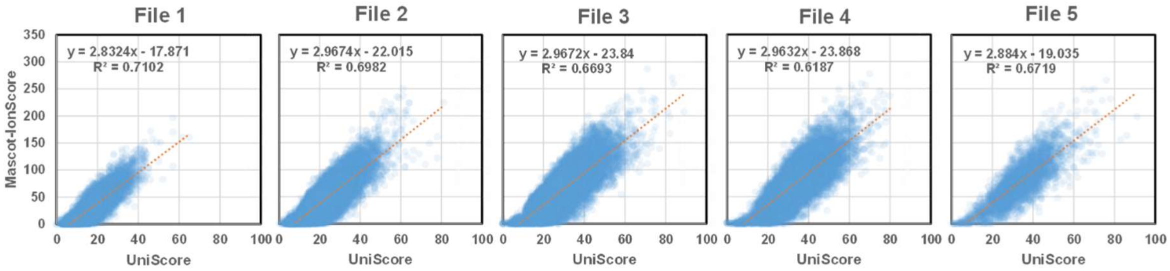

Supplementary Figure S3: Correlation between UniScore and Mascot ion score for peptides with different physical properties in Files 1-5.

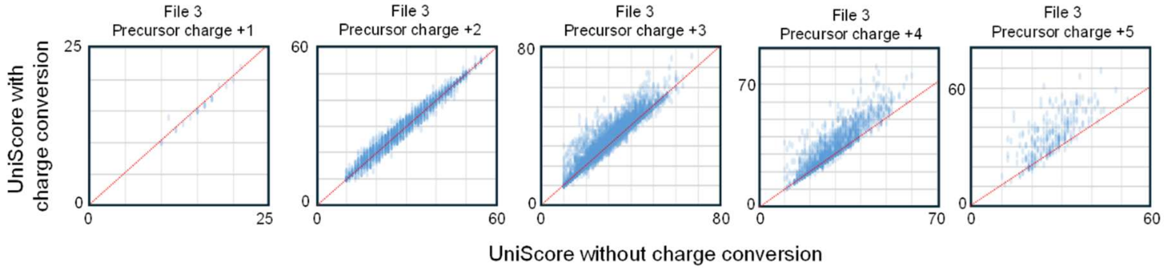

Supplementary Figure S4: Effect of charge conversion on UniScore of peptides with different precursor charges. The red dotted line shows y = x.

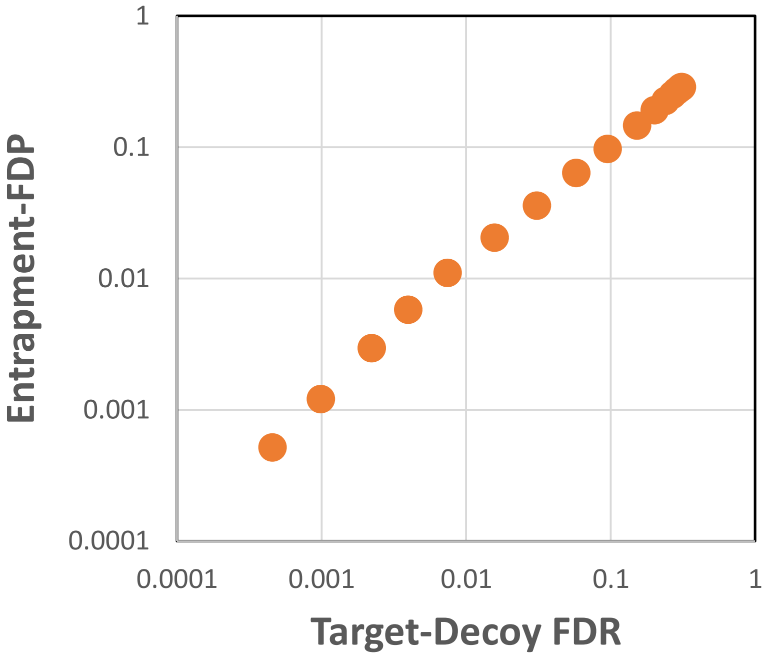

Supplementary Figure S5: Decoy-based FDR using linear discriminant analysis is well-calibrated against entrapment-based FDP.

Supplementary Table S1: Datasets and data analysis parameters used in this study

Datasets		
Dataset #1<br>for UniScore development	Original ID	PXD005159
	Reanalysis ID	RPXD034294/ JPST001624
Dataset #2<br>for UniScore application<br>(global proteomics)	Original ID	PXD004452
	Reanalysis ID	RPXD056882/ JPST003443
Dataset #3<br>for UniScore application<br>(phosphoproteomics)	Original ID	PXD018357
	Reanalysis ID	RPXD034696/ JPST001780
**Search engines and other tools with version**		
Mascot	2.7.0	
MaxQuant:	1.6.17.0（Download date: 2021/01/26）	
Comet	2019.01 rev. 5	
X! Tandem	2015.04.01.1	
Percolator	v3-04	
ProteoWizard	3.0.21021.e2078b75f 64-bit（Download date:2021/01/25)	
**Reanalysis search parameters**		
Database<br>Enzyme<br>Fixed modifications<br>Variable modifications<br>MS1 tolerance<br>MS2 tolerance<br>Missed cleavage	Listed in jPOST repository (https://repository.jpostdb.org/) with RPXD identifiers	
Input peak list	mgf files generated by MaxQuant	
Peak matching for UniScore	Top 12 peaks within every 100 Th bin	

Supplementary Table S2: Effect of product ion abundance filtering on UniScore-PSM at FDR 1%

Bin size (Th)	30			50							100								
Top N/bin	3	4	5	4	5	6	6	7	8	10	6	8	10	11	**12**	12	13	14	15
Product ion charge	1	1	1	1	1	1	1,2	1	1	1	1	1	1	1	**1**	1,2	1	1	1
File 1	7,901	8,253	7,972	8,199	8,084	8,266	7,909	7,911	7,995	8,080	7,654	7,765	8,114	8,228	**8,296**	7,961	8,347	8,383	8,404
File 2	23,257	23,769	23,445	22,995	23,530	23,767	23,823	23,898	23,465	23,576	22,048	23,189	23,669	23,768	**23,840**	23,353	23,350	23,411	23,446
File 3	21,898	21,862	21,887	21,853	21,926	21,943	21,874	21,879	21,889	21,897	21,739	21,882	21,926	21,935	**21,947**	21,875	21,873	21,879	21,885
File 4	14,400	14,404	14,408	14,390	14,399	14,405	14,410	14,407	14,408	14,409	14,383	14,397	14,401	14,405	**14,405**	14,409	14,406	14,406	14,408
File 5	3,290	3,290	3,290	3,290	3,290	3,291	3,292	3,291	3,291	3,291	3,288	3,288	3,289	3,290	**3,291**	3,292	3,291	3,291	3,291
Total	70,746	71,578	71,002	70,727	71,229	71,672	71,308	71,386	71,048	71,253	69,112	70,521	71,399	71,626	**71,779**	70,890	71,267	71,370	71,434

Red bold numbers indicate the maximum number of PSMs in each row. Search parameters as listed in Supple Table 1 except the parameters in top N in bin X Th

Supplementary Table S3: Effect of matched b-, y-ions on UniScore-PSM at FDR 1%

Coefficient for (b and y) sites x 2	0	0.1	0.2	0.3	0.4	**0.5**	0.6	0.7	0.8	0.9	1
Coefficient for (b or y) sites	1	0.9	0.8	0.7	0.6	**0.5**	0.4	0.3	0.2	0.1	0
File 1	1,270	1,616	3,078	5,241	7,442	**7,544**	6,067	4,439	3,650	3,528	3,143
File 2	8,359	11,315	15,403	19,413	23,195	**23,426**	20,988	18,296	15,158	14,603	10,723
File 3	14,576	17,022	20,985	22,758	24,254	**24,687**	23,752	21,676	18,229	16,598	14,582
File 4	14,629	16,280	16,848	17,137	**17,161**	17,143	16,958	16,301	14,927	13,253	11,023
File 5	3,931	4,021	4,053	**4,073**	4,069	4,061	4,020	3,824	3,200	2,333	1,890
Total	42,765	50,254	60,367	68,622	76,121	**76,861**	71,785	64,536	55,164	50,315	41,361

Red bold numbers indicate the maximum number of PSMs in each row.

Supplementary Table S4: UniScore-based PSMs at FDR 1% (target-decoy) and FDP 1% (entrapment).

	UniScore	
	Target-decoy FDR 1%	Entrapment FDP 1%
File 1	8,235	8,853
File 2	25,229	25,828
File 3	25,066	25,209
File 4	17,132	17,136
File 5	4,063	4,070
Total	79,725	81,096

Supplementary Table S5: Various Search Engines, UniScore and Quantms for PSM at FDR 1%.

	MaxQuant	MSFragger 4.1	Sage 0.14.7	MaxQuant/Comet/XTandem/Mascot			Comet/Sage/MSGF+		
				UniScore	UniScore-PCL	Quantms	UniScore	UniScore-PCL	Quantms
File 1	7,078	8,253	8,070	8,235	8,853	7,097	6,988	8,192	6,857
File 2	19,742	21,274	21,593	25,229	25,828	22,031	23,545	25,183	20,070
File 3	19,232	20,209	20,290	25,066	25,209	22,301	24,395	24,933	21,968
File 4	13,080	14,339	14,368	17,132	17,136	15,651	16,589	16,747	15,016
File 5	2,897	3,424	3,503	4,063	4,070	3,837	3,865	3,900	3,185
Total	62,029	67,499	67,824	79,725	81,096	70,917	75,382	78,955	67,096

Supplementary Table S6: Estimation of UniScore Calculation

- CPU — Intel Xeon Gold 6248R @ 3.00GHz
- memory — 512GB
- OS — Windows Server 2016 Standard
- programming language — Ruby 2.7 x64
- UniScore calculation process — Single thread coding

ID	File name	Raw file size	mgf size	UniScore computation time (h:m:s)
JPST000200	150211tk04-whole_2m8h-1.raw	1.3G	160M	0:01:50
	150211tk04-whole_2m8h-2.raw	1.1G	269M	0:04:14
	150211tk04-whole_2m8h-3.raw	974M	194M	0:03:12
	150211tk04-whole_2m8h-4.raw	681M	111M	0:02:02
	150211tk04-whole_2m8h-5.raw	578M	40M	0:00:31
